# Strategies for reopening in the forthcoming COVID-19 era in China

**DOI:** 10.1093/nsr/nwac054

**Published:** 2022-04-06

**Authors:** Wei-jie Guan, Nan-shan Zhong

**Affiliations:** State Key Laboratory of Respiratory Disease, National Clinical Research Center for Respiratory Disease, Guangzhou Institute of Respiratory Health, The First Affiliated Hospital of Guangzhou Medical University, Guangzhou Medical University, China; Department of Thoracic Surgery, Guangzhou Institute for Respiratory Health, The First Affiliated Hospital of Guangzhou Medical University, Guangzhou Medical University, China; Department of Respiratory and Critical Care Medicine, Foshan Second People's Hospital, Affiliated Foshan Hospital of Southern Medical University, China; State Key Laboratory of Respiratory Disease, National Clinical Research Center for Respiratory Disease, Guangzhou Institute of Respiratory Health, The First Affiliated Hospital of Guangzhou Medical University, Guangzhou Medical University, China

The transmission of severe acute respiratory syndrome coronavirus-2 (SARS-CoV-2), the culprit pathogen of coronavirus disease 2019 (COVID-19), has resulted in more than 0.43 billion cases and 6 million deaths globally. Compared with other existing variants of concern, the Omicron variant is characterized by significantly greater infectivity (Ro ≈ 6), greater tropism of the upper respiratory tract and lower severity of human infections [1]. These characteristics of the Omicron variant are the basis upon which several developed countries are considering reopening.

Different policies have been adopted worldwide during the COVID-19 era. The community-based dynamic zeroing policy has played a pivotal role in minimizing the number of laboratory-confirmed cases and deaths in mainland China. The rapid roll-out of vaccinations, as well as herd immunity, have formed the fundamentals of the ‘total opening’ of some developed countries. This, however, does not necessarily justify rapid and total opening in mainland China. Omicron confers a considerably higher mortality risk than seasonal influenza, and would likely result in social instability and the emergence of other novel variants with a greater infectivity.

The dynamic zeroing policy has been adopted for maintaining effective disease prevention and control. However, China needs to reopen so as to normalize socio-economic development and adapt to global reopening. Prolonged dynamic zeroing cannot be pursued in the long run. There are several recommendations on how China could reopen in an orderly and effective manner.

First, enforcing nationwide vaccination is crucial to safeguarding herd immunity. As of 28 February 2022, China has vaccinated 87.64% of the whole population with at least two doses of SARS-CoV-2 vaccines, with 40.32% completing the third-dose boosting vaccination. However, the vaccination rate was still markedly lower than 83% in the Chinese population aged >70 years in mainland China and even lower in Hong Kong [2]. The full dose vaccination among over 80% of the population was associated with a markedly reduced mortality rate [2]. Homologous boosting with a third dose of BioNTech vaccine significantly increased the neutralizing antibody titers against the Omicron variant [3]. Two doses of an inactivated SARS-CoV-2 vaccine (CoronaVac, Sinovac Biotech) conferred protection against the Delta variant [4], and an analysis of the protective effects of the third dose of homologous CoronaVac vaccines on the Omicron variant is underway. Furthermore, recent laboratory data have demonstrated that heterologous boosting of the two-dose CoronaVac vaccine with either the BNT162b2 (Comirnaty, Pfizer-BioNTech) vaccine, the recombinant protein subunit vaccine (ZF2001, Zhifei Longcom Biopharmaceutical) [5] or the adenovirus vaccine (CanSino Biologics) could markedly enhance protection against the Omicron variant. These findings have lent support to the heterologous boosting strategy intended to increase the rate of protection against variants of concern (particularly the Omicron variant).

Second, efforts to reduce the risk of progression to critical illness and death would benefit from the acceleration of research and development of targeted medications and potent neutralizing antibodies. In addition to paxlovid and molnupiravir, the therapeutic effects of medications (e.g. VV116) and neutralizing antibodies (BRII-196 and BRII-198) have provided hope with regard to improving clinical outcomes, although conducting phase three studies within mainland China would be challenging due to limited patient sources. International collaboration should therefore be encouraged.

Third, rapid antigen testing should be prioritized in community settings. Antigen testing has been associated with significantly shorter turnaround times and less reliance on medical facilities and personnel compared to nucleic acid testing. This ensures timely identification of the source patient, on which basis population-based screening with viral nucleic acid testing can be reserved for close contacts (particularly the imported cases).

Fourth, we should strengthen longitudinal follow-up investigations of infected cases during the latency and convalescent periods. In-depth analysis of the infectivity of imported cases (including those showing re-positive test results) would inform the policy maker with regard to the minimal duration of quarantine and the management of re-positive cases after hospital discharge.

Finally, performing pilot investigations in certain designated cities or regions, as well as adjusting the policy according to the epidemic characteristics of imported cases, will be equally vital for verifying the outcomes of the transition towards safe and orderly social reopening in China.

## Supplementary Material

nwac054_Supplemental_File
