# Supplementary material for: Strategies for reopening in the forthcoming COVID-19 era in China
Source: Natl Sci Rev. 2022 Apr 6;9(3):nwac054. doi: 10.1093/nsr/nwac054 (PMC8986455; doi:10.1093/nsr/nwac054)
Supplement: nwac054_Supplemental_File [file nwac054_supplemental_file.doc]

**Strategies for re-opening of China at the forthcoming COVID-19 era**

Wei-jie Guan^1,2^, Nan-shan Zhong^1^

1. State Key Laboratory of Respiratory Disease, National Clinical Research Center for Respiratory Disease, Guangzhou Institute of Respiratory Health, The First Affiliated Hospital of Guangzhou Medical University, Guangzhou Medical University, Guangzhou, China
2. Department of Thoracic Surgery, Guangzhou Institute for Respiratory Health, The First Affiliated Hospital of Guangzhou Medical University, Guangzhou, China

**Running head:** Strategies for re-opening in China

**Acknowledgment**: We thank Kai-ping Li for her help with the material for this editorial.

**Funding**: Supported by Emergency Key Program of Guangzhou Laboratory (Grant No. EKPG22-2) and Guangzhou Institute for Respiratory Health Open Project (funded by China Evergrande Group) - Project No. 2020GIRHHMS09 and 2020GIRHHMS19.

**Author’s contribution:** W. J. G. and N. S. Z. drafted the manuscript; both authors provided critical review of the manuscript and approved the final draft for publication.

**Conflict of interest:** None declared.
